# Reversible strain-induced magnetization switching in FeGa nanomagnets: Pathway to a rewritable, non-volatile, non-toggle, extremely low energy straintronic memory

**DOI:** 10.1038/srep18264

**Published:** 2015-12-14

**Authors:** Hasnain Ahmad, Jayasimha Atulasimha, Supriyo Bandyopadhyay

**Affiliations:** 1 Dept. of Electrical and Computer Engr., Virginia Commonwealth University, Richmond, VA 23284, USA; 2 Dept. of Mechanical and Nuclear Engr. Virginia Commonwealth University, Richmond, VA 23284, USA

## Abstract

We report reversible strain-induced magnetization switching between two stable/metastable states in ~300 nm sized FeGa nanomagnets delineated on a piezoelectric PMN-PT substrate. Voltage of one polarity applied across the substrate generates compressive strain in a nanomagnet and switches its magnetization to one state, while voltage of the opposite polarity generates tensile strain and switches the magnetization back to the original state. The two states can encode the two binary bits, and, using the right voltage polarity, one can write either bit deterministically. This portends an ultra-energy-efficient non-volatile “non-toggle” memory.

Nanomagnets are the staple of non-volatile memory. Binary bit information (‘0’ or ‘1’) is stored in a stable or metastable magnetization state. Writing a bit involves switching the magnetization to the desired state by an external agent. This process should be minimally dissipative and should not require knowledge of the earlier stored bit to write the desired bit. That is, the writing agent should not have to read the earlier stored bit first and then decide on a course of action to write the desired bit based on that knowledge. Memory which lacks the latter feature is called “toggle” memory. There, the writing agent first reads the previously stored bit and if it is the same as the desired bit, does nothing. If it is not, then it toggles the stored bit to write the desired bit^1^,^2^. A superior scheme is “non-toggle” memory where the writing cycle is not preceded by a reading cycle. The writing agent can write either bit deterministically without needing to know what the previously stored bit was.

There are many schemes for altering the magnetization state of a nanomagnet to enable the writing of a bit. The oldest is to use a local magnetic field generated by an on-chip current, which is, regrettably, extremely dissipative^3^ and would dissipate about 10^7^ kT of energy at room temperature per write operation^4^. The second is to use a spin-polarized current to deliver a spin-transfer torque^5^ or induce domain wall motion^6^. These are also extremely dissipative strategies and dissipate between 10^4^ kT and 10^7^ kT of energy per write step^7^,^8^. The recent use of the giant spin Hall effect to generate a spin polarized current^9^ may end up reducing the energy dissipation to perhaps ~10^4^ kT per step, but a much more energy-efficient approach is to use two-phase multiferroics (a magnetostrictive nanomagnet delineated on a piezoelectric film). An electrostatic potential applied across the piezoelectric film generates strain in that layer, which is partially transferred to the magnetostrictive layer and rotates the latter’s magnetization via the Villari effect. Non-toggle writing schemes based on such strain-induced switching of a magnetostrictive nanomagnet from one stable state to another have been proposed in the past^10^,^11^. Electrostatic potential of one polarity generates compressive strain in the nanomagnet and switches the magnetization to one state (to write bit ‘0’) and voltage of the opposite polarity generates tensile strain and switches the magnetization to the other state (to write bit ‘1’). This is illustrated in Fig. 1(a–c). According to theoretical calculations, roughly 850 kT of energy will be dissipated to write a bit in ~1.5 ns at room temperature in this type of non-toggle memory^11^.

There are a number of reports of switching the magnetization of nanoscale two-phase multiferroics with electrically generated strain^12^,^13^,^14^. There is also a report of switching the resistance of a magneto-tunneling junction (MTJ) whose soft layer is a two-phase multiferroic (CoFeB/PMN-PT) with electrically generated strain [the MTJ is a complete memory element with read/write], but the MTJ had tens of *μ*m-scale dimensions (not nanoscale)^15^. Most important, all of the above experiments employed ferromagnets with low saturation magnetostriction (Co, Ni, CoFeB), which is not conducive to energy efficiency since the stress needed to rotate the magnetization (and correspondingly the voltage needed to generate the stress) is inversely proportional to the magnetostriction coefficient of the magnetostrictive component. In this work, we have used FeGa as the magnetostrictive component of the nanoscale multiferroic. Because FeGa has a much higher magnetostriction (approximately an order of magnitude higher in bulk) than Co or Ni, the electric field needed to alter its magnetization is correspondingly smaller. There are previous demonstrations of magnetization rotation in FeGa layers with strain^16^,^17^,^18^,^19^, but the samples were, again, not nanoscale, except in^20^ which did not demonstrate repeatable switching. Even more important, none of the above experiments addressed, let alone demonstrate, the *“non-toggle” behavior*, i.e., the magnetization being driven to one state with one sign of stress/strain and restored back to the other (original) state with the opposite sign of stress/strain. Here, we have achieved this feat.

Elliptical FeGa nanomagnets of major axis ~300 nm, minor axis ~240 nm, and thickness ~8 nm were fabricated on a (100)-oriented PMN-PT substrate (70% PMN and 30% PT). Details of fabrication can be found in the Methods section and further details of characterization can be found in ref. 20. The substrate was first poled with an electric field of 8400 V/cm in a direction which will coincide with the major axes of the nanomagnets (the major axes of the nanomagnets were nominally parallel to each other) prior to fabrication of the nanomagnets.

The sputtered FeGa films constituting the naomagnets were characterized with x-ray diffraction (XRD) and layer-by-layer x-ray photoelectron spectroscopy (XPS) while etching off layers with an Ar ion beam. The XRD revealed that the film is polycrystalline with mostly (110) textured growth, while the XPS showed that the alloy composition was different in different layers with some incorporation of oxygen in the topmost layers due to ambient oxidation^20^. The magnetization (M-H) curves of FeGa layers were measured at 77 K and 300 K in a vibrating sample magnetometer and showed not only ferromagnetic behavior, but that the layer had in-plane magnetic anisotropy^20^. The measured in-plane coercivity was ~180 Oe and the out-of-plane coercivity was ~120 Oe^20^. Interestingly, the M-H curves showed “shoulders”^20^ indicative of the presence of more than one phase in FeGa, each with a different coercivity, as previously noted in other materials^21^. This indicated the presence of multiple energy barriers in the potential profiles of the FeGa nanomagnets which could result in the formation of metastable magnetization states. Stress could always drive a nanomagnet into such a state where it will remain after stress is withdrawn since the state is “metastable” and robust against thermal perturbations at room temperature. Metastable magnetization states could, of course, arise from other effects as well, such as due to pinning sites or irregular geometry of the nanomagnets caused by imperfect electron-beam lithography. There is a recent report of non-Joulian magnetostriction in FeGa^22^ which could further complicate the nanoscale switching. In the grand scheme, none of this is important; all that matters is that stress of one sign drives the magnetization from one distinct state to another, where it stays after withdrawal of stress, and then stress of the opposite sign brings the magnetization back to the original state. This will result in a non-volatile, non-toggle, rewritable, straintronic memory.

In order to study magnetization switching, we first magnetized all FeGa nanomagnets on a PMN-PT wafer with a ~2 Tesla magnetic field directed along the nominal major axis of the nanomagnets. The magnetization states of nanomagnets were then determined with magnetic force microscopy (MFM). Care was taken to use low-moment tips in order to not perturb the magnetization states of the nanomagnets with the tip. MFM imaging showed that (see second vertical panels of Figs 2 and 3) most nanomagnets have been magnetized in the direction of the field, but some have been magnetized in an orientation that subtends a non-zero angle with the magnetizing field. This odd behavior is ascribed to the presence of spurious energy minima in the potential profile of these nanomagnets in a magnetic field that are caused by either the presence of multiple energy barriers due to irregular shapes, or multiple phases, or pinning sites. The magnetic field drives a nanomagnet to an energy minimum closest to the initial state, whose magnetization orientation may not be collinear with the field.

After the initial magnetizing, every nanomagnet is *compressively* stressed along its major axis by subjecting the PMN-PT substrate to a global average electric field of 4.4 kV/cm in a direction opposite to that of the initial poling (see Fig. 1(d)). The field is generated by applying a potential of −2.2 kV along a 5 mm long substrate. It strains the PMN-PT substrate owing to *d*_33_ coupling. The value of 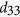 measured in our substrates in ref. 14 was 1000 pm/V. Therefore, the average strain generated in the PMN-PT substrate is 440 ppm. This strain is partially or completely transferred to the FeGa nanomagnets, resulting in a maximum stress of ~33 MPa in the nanomagnets since the Young’s modulus of FeGa is about 75 GPa^23^. The electric field lines in the vicinity of the nanomagnets are not necessarily directed along the major axes because of fringing effects and the stress on a nanomagnet is not necessarily uniaxial along the major axis. However, the exact field or stress distribution in space is not important; in the end, the *average* stress generated in a nanomagnet is either compressive or tensile depending on the polarity of the voltage. This compression or tension alters the magnetization states.

Compressive stress makes the magnetization evolve to a new state, and the magnetization stays there after the stress (electric field) is removed, showing that the new state is “non-volatile”. This is shown in the third vertical panels of Figs 2 and 3. Next, *tensile* stress is applied along the major axis of the nanomagnets by reversing the polarity of the voltage from −2.2 kV to + 2.2 kV. The fourth vertical panels of Figs 2 and 3 show that all nanomagnets that were imaged returned to the original state after experiencing tension (it is possible, however, that some nanomagnets that were not imaged failed to return to their original states). All this shows two important features: First, one can “rewrite” a bit in the nanomagnets after the first writing (rewritable non-volatile memory), and second, the switching is “non-toggle”. Writing does not always require toggling the previously stored bit; if we apply a positive voltage and tensile stress, then we will always deterministically write the bit 1 irrespective of whether the previously stored bit was 1 (no toggling required) or 0 (toggling required). The same is true if we wish to write the bit 0. In fact, we do not even need to know what the previously stored bit was, which avoids a read step.

In Fig. 4, we show magnetic force micrographs of three FeGa nanomagnets to establish that the stress-induced magnetization switching between two distinct states is *repeatable*. In successive vertical panels, we present the initial magnetization state, the state after the first compression cycle, the state after the first tension cycle, the state after the second compression cycle, and the state after the second tension cycle. In the nanomagnets that switch, compression always drives the magnetization away from its pre-stress state and tension always brings it back to the pre-stress state. Unfortunately, we cannot cycle the stress too many times to assess the endurance since the piezoelectric substrate develops fatigue and physically cracks after a few cylces. Our substrate is sourced from a commercial vendor and not of the quality of thin films. Unclamped thin films grown on high quality substrates^24^ will presumably be less prone to fatigue and exhibit better behavior. Although, we cannot test the endurance, we note that the effect is perfectly repeatable and is seen in every nanomagnet that shows the switching behavior. We show the MFM images for three arbitrarily chosen nanomagnets and all three exhibit this feature.

One important observation is that the state under tension is unique, but the state under compression is not. At different times, compression drives the magntization to different states. This is not surprising in a multi-phase ferromagnet where there will be multiple metastable states and different ones can be accessed at different times when the nanomagnet is compressed. Which of these states is visited under compression is not controllable, but what is important is that the visited state *is always distinct from the state visited under tension*. For memory applications that is sufficient.

We will call the state under tension “logic bit 0” and the state under compression “not logic bit 0” or “something different from 0”, as opposed to calling it “logic bit 1”. For memory applications, only one of the states needs to be unique; the other state need not be unique. Here state ‘0’ is unique and whenever that state is read by the reader, we will determine that the stored bit is 0. Whenever that state is *not read*, we will determine that the stored bit is ‘not 0’. In binary memory, anything that is ‘not 0’ can be interpreted as ‘1’ as long as the state ‘0’ is unique. This will not work for logic where both bits have to be encoded in unique states since logic gates are concatenated (unlike memory cells which are isolated) and the output of one gate feeds into the input of the next. That is why one requires level restoration in logic, but not in memory. Here, we are claiming only memory application, for which only one state needs to be unique and that is indeed the case.

We could fashion a memory element by using a magneto-tunneling junction (MTJ) with an FeGa soft layer and magnetize the hard layer permanently in a direction anti-parallel to the pre-stress magnetization orientation of the soft layer. Then, tensile stress applied with one voltage polarity will always take the MTJ resistance to a high state (encoding, say, bit 1) and compressive stress applied with the other voltage polarity will take the MTJ resistance to a different state encoding the logic complement of the bit 1. In the supplementary material, we show a possible implementation for a memory array with isolation between memory cells and read/write lines. We also describe the read/write strategy.

In conclusion, we have demonstrated the core component of a *straintronic* non-volatile, rewritable, non-toggle memory element. Since the magnetization switching is induced by strain, it should be remarkably energy-efficient going by all available theoretical predictions^11^,^25^,^26^,^27^. Here, we unfortunately had to apply a global strain with a large voltage across a large substrate (5 mm wide) because delineating local contact pads around each nanomagnet to apply the field locally was lithographically too challenging for us. However, such lithography is not challenging for a fabrication foundry. If the electrode separation is reduced to 500 nm from 5 mm, then the voltage would reduce by a factor of 5 mm/500 nm = 10,000 times and the energy dissipation (proportional to the square of the voltage) would reduce by 10^8^ times.

Strain can be localized in a piezoelectric thin film with appropriately placed gate pads^28^. This provides isolation between bits. Alternately, one can mesa-etch the piezoelectric film and delineate a nanomagnet on each mesa for bit isolation. Mesa-etching a piezoelectric film may or may not degrade the piezoelectric property, but this is a material issue that needs to be addressed separately. In the supplementary material, we show two different schemes for a memory array compatible with a crossbar architecture that involve bit isolation effected by either gate pads or mesa etching.

Consider the memory array shown in the figure in the supplementary material. If strain is generated in a nanomagnet in the manner of, say, ref. 24, then a voltage will be applied between the top contacts and the grounded substrate to strain the nanomagnets. In that case, the electric field of 4.4 kV/cm will appear across the thickness of a ~100 nm piezoelectric layer, resulting in a switching voltage *V* of only 4.4 kV/cm × 100 nm = 44 mV. This method generates biaxial strain (tension along the minor axis of the elliptical nanomagnets and compression along the major axis, or vice versa, depending on the voltage polarity^24^) which is even better because it results in larger stress anisotropy energy. This may further reduce the voltage (and electric field) needed to rotate magnetization and therefore further reduce the energy dissipation.

For an effective electrode area of 100 nm × 100 nm and a piezoelectric film thickness of 100 nm, the electrode capacitance in the scheme of^24^ would be ~2 × 1000 × 8.854 × 10^−12^ × 100 nm × 100 nm/100 nm = 1.7 fF^1^ (there are two electrodes that are shorted in the scheme of^24^). The resulting energy dissipation 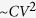 to write a bit would have been only 1.7 fF × (0.044 V)^2^ = 3.3 aJ (788 kT at room temperature). There is also some internal energy dissipation in the nanomagnet owing to Gilbert damping, but that is on the order of 1 aJ^1^. If we add that, the total dissipation would be ~4.3 aJ (1027 kT). In contrast, present day mainstream spin-transfer-torque random access memory (STT-RAM) dissipates about 10^7^ kT of energy per write operation^8^ and spin-Hall based versions will dissipate ~10^4^ kT. Therefore, this experiment lays the foundation of a remarkably energy-efficient non-toggle non-volatile straintronic memory technology.

## Methods

Poled PMN-PT substrates were spin-coated with two layers of PMMA (e-beam resist) of different molecular weights in order to obtain superior nanomagnet feature definition: PMMA-495 Anisol and PMMA-950 Anisol at 2500 rpm spinning rate. The resists were baked at 115 Celsius for 2 minutes and exposed in a Hitachi SU-70 SEM with a Nabity attachment using 30 kV accelerating voltage and 60 pA beam current. Subsequently, the resists were developed in MIBK:IPA (1:3) for 90 seconds followed by rinsing in cold IPA.

For nanomagnet delineation, a 3–3.5 nm thick Ti adhesion layer was first deposited using e-beam evaporation at a base pressure of (2–3) × 10^−7^ Torr, followed by the deposition of 7–8 nm of FeGa (thickness verified with AFM) using DC magnetron sputtering of a FeGa target with a base pressure of (2–3) × 10^−8^ Torr and deposition pressure of 1 mTorr. The magnetron power was 45 W and the deposition was carried out for 40 seconds.

## Additional Information

**How to cite this article**: Ahmad, H. *et al.* Reversible strain-induced magnetization switching in FeGa nanomagnets: Pathway to a rewritable, non-volatile, non-toggle, extremely low energy straintronic memory. *Sci. Rep.* **5**, 18264; doi: 10.1038/srep18264 (2015).

## Supplementary Material

Supplementary Information

## Figures and Tables

**Figure 1:**
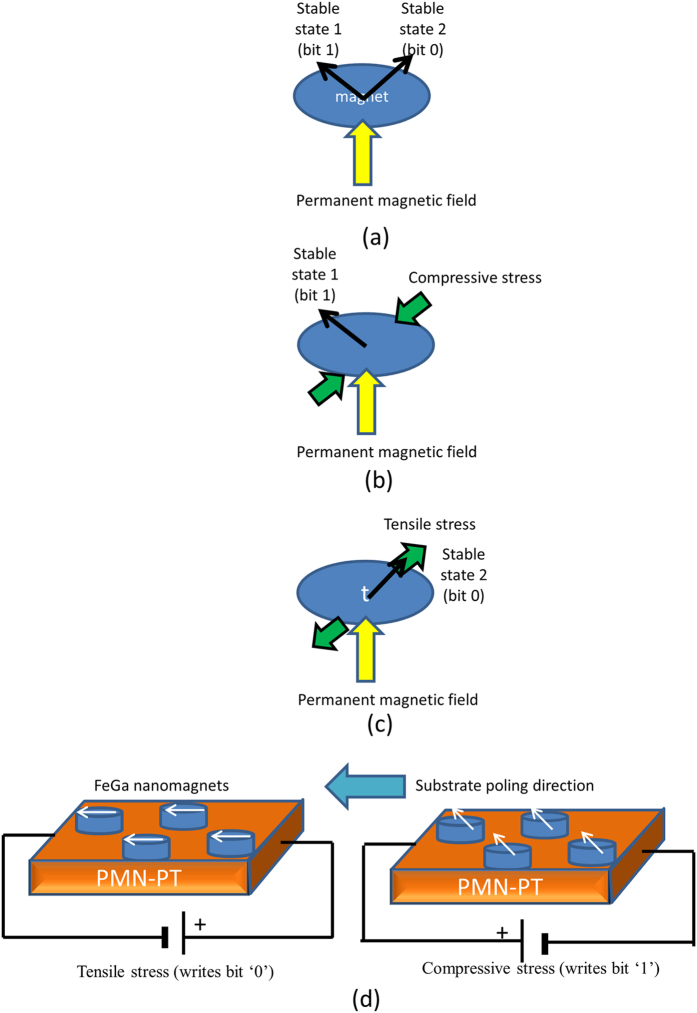
(**a**–**c**) Non-toggle straintronic memory of the type discussed in refs 10,11. (**a**) A magnetic field applied along the minor axis of an elliptical nanomagnet gives rise to two stable magnetization orientations at an angle with each other (the angle depends on the magnetic field strength, shape of the nanomagnet, etc.). They encode bits ‘0’ and ‘1’. (**b**) We will assume that the magnetostriction coefficient of the nanomagnet is positive. Then, compressive stress along an axis collinear with one stable state (say, state 2) causes the magnetization to settle into state 1. If the magnetostriction coefficient were negative, the magnetization would have settled into state 2. (**c**) Tensile stress along the same axis causes the magnetization to settle into state 2 for positive magnetostriction and state 1 for negative magnetostriction. Therefore, we can write either bit by choosing the sign of the stress along the stress axis. We do not need to know what the previously stored bit was in order to write the desired bit. (**d**) Our test set-up. Electric field in the direction of substrate poling generates tensile stress in the nanomagnets whose major axes are aligned collinear with the poling direction. This aligns the magnetizations along one direction and writes bit ‘0’. Electric field in the opposite direction generates compressive stress and aligns the magnetizations in a different direction, writing bit ‘1’.

**Figure 2:**
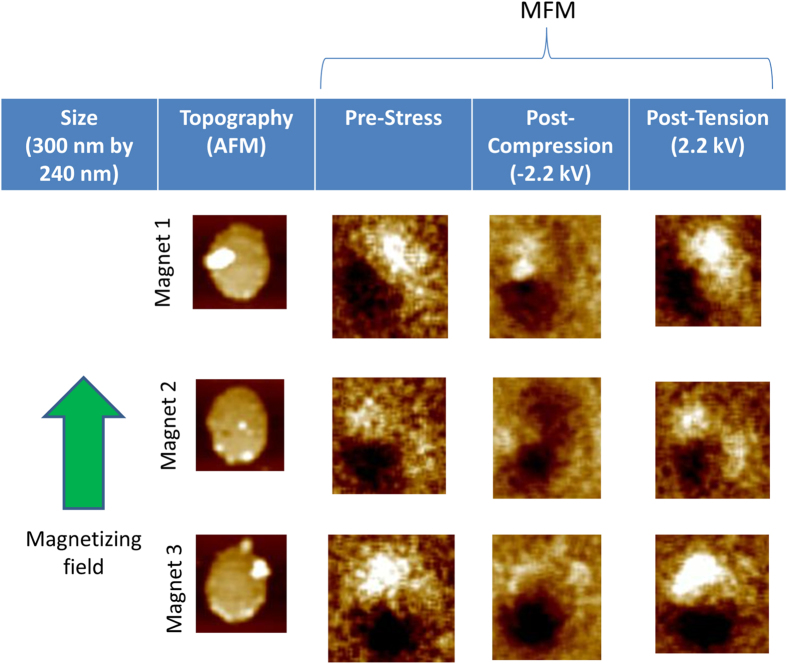
Magnetic force (MFM) and atomic force (AFM) micrographs of three isolated elliptical FeGa nanomagnets that have all been magnetized with a magnetic field in the direction indicated by the thick vertical green arrow. Starting from the left, the first vertical panel shows the AFM image of the nanomagnet, the second vertical panel shows the initial magnetization state after magnetizing with the field (note that the magnetization is not always in the direction of the field), the third vertical panel shows the new magnetization state after compressive stress is applied and withdrawn, and the last vertical panel shows the magnetization state after tensile state is applied and withdrawn. Note that compressive stress takes the magnetization to a state different from the initial one and keeps it there after stress withdrawal (non-volatile). Tensile stress brings it back to the original state and keeps it there after stress withdrawal. Thus, tensile stress always writes the bit ‘0’ and compressive stress writes a bit that is ‘not-0’ and we call it bit ‘1’. This realizes a non-volatile, non-toggle memory.

**Figure 3:**
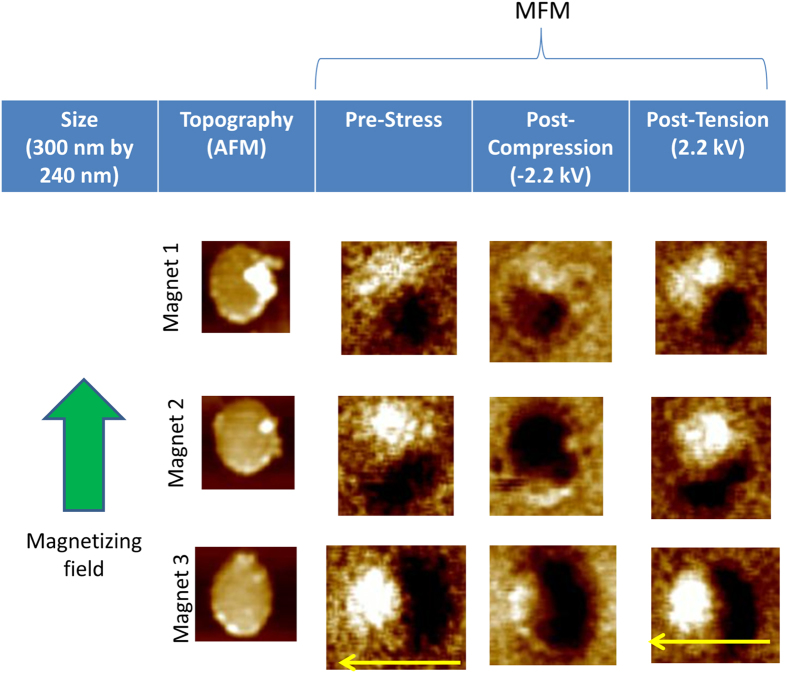
Same as Fig. 2, but for a different set of nanomagnets. Here, the nanomagnet in the last row was magnetized in a direction almost perpendicular to the magnetizing field showing that there is a deep energy minimum corresponding to that orientation and the nanomagnet prefers to go there even in the presence of the magnetizing field. Compressive stress, however, seems to drive it out of that state, but subsequent application of tensile stress brings it back to that state, just like in the case of the other nanomagnets. Again, a non-volatile, non-toggle memory is implemented.

**Figure 4:**
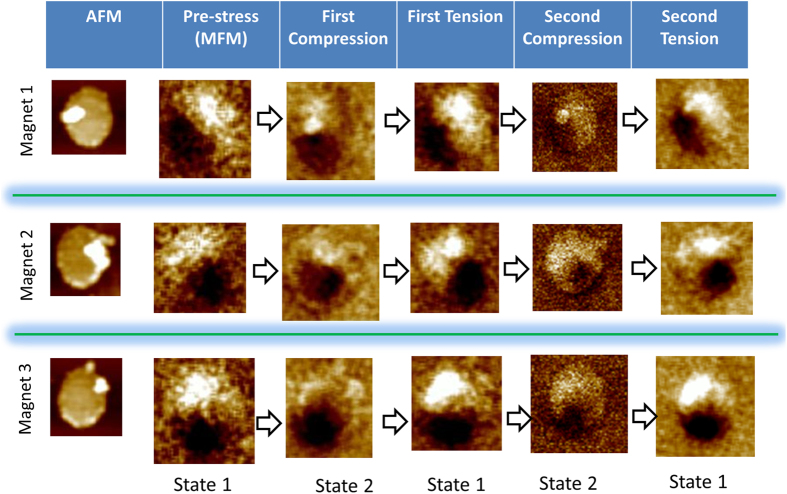
Magnetic force microscope images of nanomagnets showing repeatability of the switching. A nanomagnet cycles through its two magnetization states repeatedly with successive compression and tension. Whenever the stress is tensile, the magnetization goes into one state and whenever stress is compressive, it goes into the other state. This consistency shows that the memory has endurance.
