# Supplementary material for: Reversible strain-induced magnetization switching in FeGa nanomagnets: Pathway to a rewritable, non-volatile, non-toggle, extremely low energy straintronic memory
Source: Sci Rep. 2015 Dec 14;5:18264. doi: 10.1038/srep18264 (PMC4677403; doi:10.1038/srep18264)
Supplement: Supplementary Information [file srep18264-s1.pdf]

Supplementary material
Reversible strain-induced magnetization switching in FeGa nanomagnets: Pathway to a rewritable, non-volatile, non-toggle, extremely low energy straintronic memory

Hasnain Ahmad[1], Jayasimha Atulasimha[2] and Supriyo Bandyopadhyay[1],
Email: {ahmadh2, jatulasimha, sbandy}@vcu.edu
[1]Dept. of Electrical and Computer Engr., [2]Dept. of Mechanical and Nuclear Engr.
Virginia Commonwealth University, Richmond, VA 23284, USA

September 30, 2015

Figure 1 shows two possible implementations of a memory array. In the first scheme, we have a continuous piezoelectric thin film and strain is localized around each nanomagnet with isolation gate pads in the manner of ref. [1]. In the second scheme, the piezoelectric layer is mesa-etched and a nanomagnet is delineated on top of each mesa. Since strain exists only in the piezoelectric material, this strategy provides automatic strain (and hence bit) isolation. The isolation gate pads are not repeatedly charged and discharged during reading/writing of bits and hence do not dissipate energy.

We follow the scheme of [2] for generating the necessary strain around each nanomagnet. The two write lines will be shorted together and a potential will be applied between them and the grounded substrate. This potential is dropped across the piezoelectric layer since the substrate is conducting. The magnitude of this potential is the product of the electric field needed to write a bit and the thickness of the piezoelectric layer, which is ∼100 nm. This potential is a few tens of mV. The piezoelectric layer is poled in the vertical direction.

Because of d_{33} and d_{31} coupling, the applied voltage generates biaxial strain around the nano-

magnets [2]. For one polarity of the voltage, there will be compression along the major axis and tension along the minor axis, whereas for the other polarity, the signs of the strain will be reversed. Thus one voltage polarity will write bit ‘0’ and the other will write bit ‘not 0’ or, equivalently, bit ‘1’ into the resistance state of the MTJ. For reading, the write lines are grounded so that there is no voltage over the piezoelectric and the MTJ resistance between the read line and the grounded write lines is read to determine if the stored bit is 0 or 1.

References

[1] Cui, J. Z., Liang, C. Y., Paisley, E. A., Sepulveda, A., Ihlefeld, J. F., Carman, G. P. & Lynch, C. S. Generation of localized strain in a thin film piezoelectric to control individual magnetoelectric heterostructures. *Appl. Phys. Lett.* **107**, 929903 (2015).

[2] Cui, J., Hockel, J. L., Nordeen, P. K., Pisani, D. M., Liang, C. Y., Carman, G. P. & Lynch, C. S. A method to control magnetism in individual strain-mediated magnetoelectric islands. *Appl. Phys. Lett.* **103**, 232905 (2013).

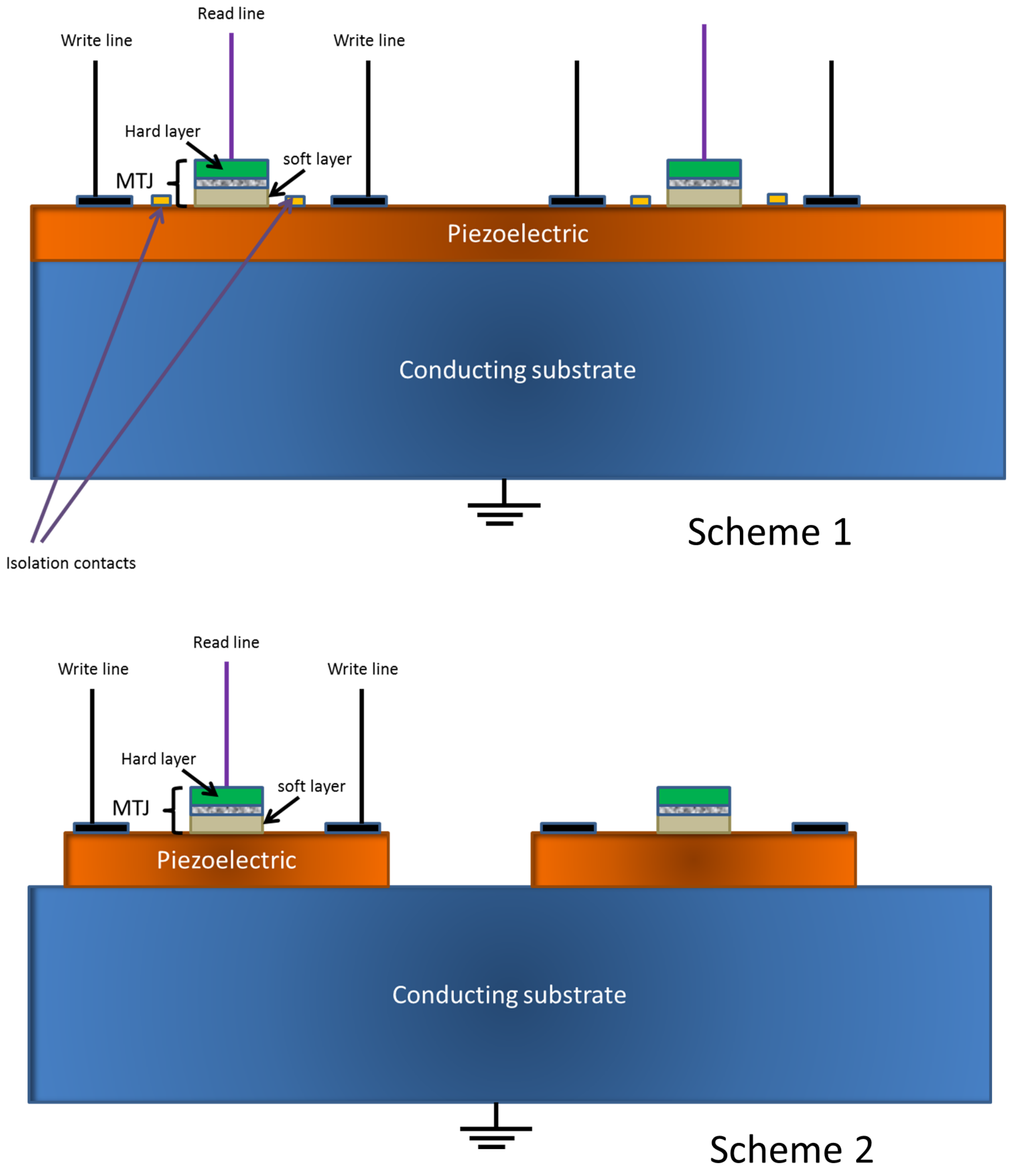

Figure 1: A straintronic memory array compatible with a crossbar architecture. Not drawn to scale.
